# Positive-charge tuned gelatin hydrogel-siSPARC injectable for siRNA anti-scarring therapy in post glaucoma filtration surgery

**DOI:** 10.1038/s41598-020-80542-4

**Published:** 2021-01-14

**Authors:** Yong Yao Chun, Zhu Li Yap, Li Fong Seet, Hiok Hong Chan, Li Zhen Toh, Stephanie W. L. Chu, Ying Shi Lee, Tina T. Wong, Timothy T. Y. Tan

**Affiliations:** 1 School of Chemical and Biomedical Engineering, Nanyang Technological University, 62 Nanyang Dr, Singapore, 637459 Singapore; 2 Ocular Imaging, Singapore Eye Research Institute, 20 College Road Discovery Tower Level 6, The Academia, Singapore, 169856 Singapore; 3 Ocular Therapeutics and Drug Delivery, Singapore Eye Research Institute, 20 College Road Discovery Tower Level 6, The Academia, Singapore, 169856 Singapore; 4 Department of Ophthalmology, Yong Loo Lin School of Medicine, National University of Singapore, 10 Medical Dr, Singapore, 117597 Singapore; 5 Duke-NUS Medical School, 8 College Rd, Singapore, 169857 Singapore; 6 Glaucoma Service, Singapore National Eye Centre, 11 Third Hospital Ave, Singapore, 168751 Singapore; 7 School of Materials Science and Engineering, Nanyang Technological University, 11 Faculty Ave, Singapore, 639977 Singapore

**Keywords:** Biomedical engineering, Drug delivery

## Abstract

Small interfering RNA (siRNA) therapy is a promising epigenetic silencing strategy. However, its widespread adoption has been severely impeded by its ineffective delivery into the cellular environment. Here, a biocompatible injectable gelatin-based hydrogel with positive-charge tuned surface charge is presented as an effective platform for siRNA protection and delivery. We demonstrate a two-step synthesis of a gelatin-tyramine (Gtn-Tyr) hydrogel with simultaneous charge tunability and crosslinking ability. We discuss how different physiochemical properties of the hydrogel interact with siSPARC (siRNA for secreted protein, acidic and rich in cysteine), and study the positive-charge tuned gelatin hydrogel as an effective delivery platform for siSPARC in anti-fibrotic treatment. Through in vitro studies using mouse tenon fibroblasts, the positive-charge tuned Gtn-Tyr hydrogel shows sustained siSPARC cellular internalization and effective SPARC silencing with excellent biocompatibility. Similarly, the same hydrogel platform delivering siSPARC in an in vivo assessment employing a rabbit model shows an effective reduction in subconjunctival scarring in post glaucoma filtration surgery, and is non-cytotoxic compared to a commonly used anti-scarring agent, mitomycin-C. Overall, the current siRNA delivery strategy involving the positive-charge tuned gelatin hydrogel shows effective delivery of gene silencing siSPARC for anti-fibrotic treatment. The current charge tunable hydrogel delivery system is simple to fabricate and highly scalable. We believe this delivery platform has strong translational potential for effective siRNA delivery and epigenetic silencing therapy.

## Introduction

Fibrosis or fibrotic scarring, which affects many organs such as skin, lung, kidney, and liver, is a leading cause of morbidity and mortality. Prevention and treatment of fibrotic diseases such as skin fibrosis (e.g. hypertrophic scars and keloids), systemic sclerosis, renal fibrosis, and post-surgical scars, remain a largely unmet clinical need. Particularly in glaucoma filtration surgery (GFS), post-operative fibrosis is common and remains the leading cause of treatment failure^1–4^. Therefore, preventing post-GFS fibrosis is vital to the overall success of glaucoma treatment. Adjunctive therapy with antimetabolites such as mitomycin-C (MMC) and 5-fluorouracil (5-FU), is regularly administered during surgery to reduce scarring^5–10^. However, these drugs have non-specific cytotoxicity, which can lead to further complications or even blindness^1,5–12^. Thus, there remains an urgent need to develop new anti-fibrotic therapeutics for post-GFS that are more specific to fibrotic targets and less toxic.

Small interference RNA (siRNA) therapy has shown potential for the prevention and treatment of fibrotic disorders. Targeting genes involved in fibrosis, such as connective tissue growth factor (CTGF/CCN-2), transforming growth factor-β (TGF-β), secreted protein, acidic and rich in cysteine (SPARC) and drosophila mothers against decapentaplegic proteins (SMADs), had been widely studied using various cellular and animal models^13–16^. Particularly for SPARC, its expression in adult tissues is frequently associated with excessive deposition of collagen, an indicator of scarring, and fibrotic disorders in a variety of organ systems such as the skin, lungs, liver, and kidneys^17–22^. In a recent study, Seet et al. demonstrated that silencing of SPARC for human tenon fibroblasts (HTF) significantly reduced pro-fibrotic genes such as collagen I and TGF-β_2_. Also, siSPARC did not affect the proliferation of HTFs and showed no cellular toxicity and apoptosis as compared to the MMC^23^. However, siRNAs can be easily degraded by RNAses^24^, and thus cannot be systemically administered for therapeutic purposes without a delivery vehicle or chemical modifications that could significantly prolong their half-lives in serum.

In another study by Seet et al., hydroxyapatite (HA) nanoparticles were used to deliver siSPARC as a promising therapeutic method for the preservation of wound filtering function in a GFS mouse model^25^. However, HA is non-resorbable and has limited biodegradability in physiological conditions^26^, which might have long-term effect on the functions of the targeted tissue. A suitable biodegradable delivery system for siSPARC is needed to prevent the disruption of tissue function. Gelatin hydrogel is a promising delivery platform for localized and sustained release of siRNA. Gelatin hydrogel is well known for being biocompatible, biodegradable, and easily processed^27^. Gelatin-based hydrogel is widely investigated as injectable system for biomedical applications^28,29^ and bio-ink for 3D printing^29,30^. Recent studies demonstrated that gelatin could be easily internalized by cells through the receptor-mediated endocytotic pathway, as it is a natural target for clathrin-type mannose receptors^31^. Gelatin also supports hemostasis and tissue restoration during wound healing^32^. However, existing gelatin hydrogel engineered for siRNA delivery fall short because of issues such as biocompatibility and ease of synthesis. They typically involve multiple synthesis steps of introducing cationic molecules (usually not biocompatible) and complex crosslinking/formation of the siRNA-hydrogel system, which might result in poor siRNA encapsulation efficiency^33–36^.

**Figure 1:**
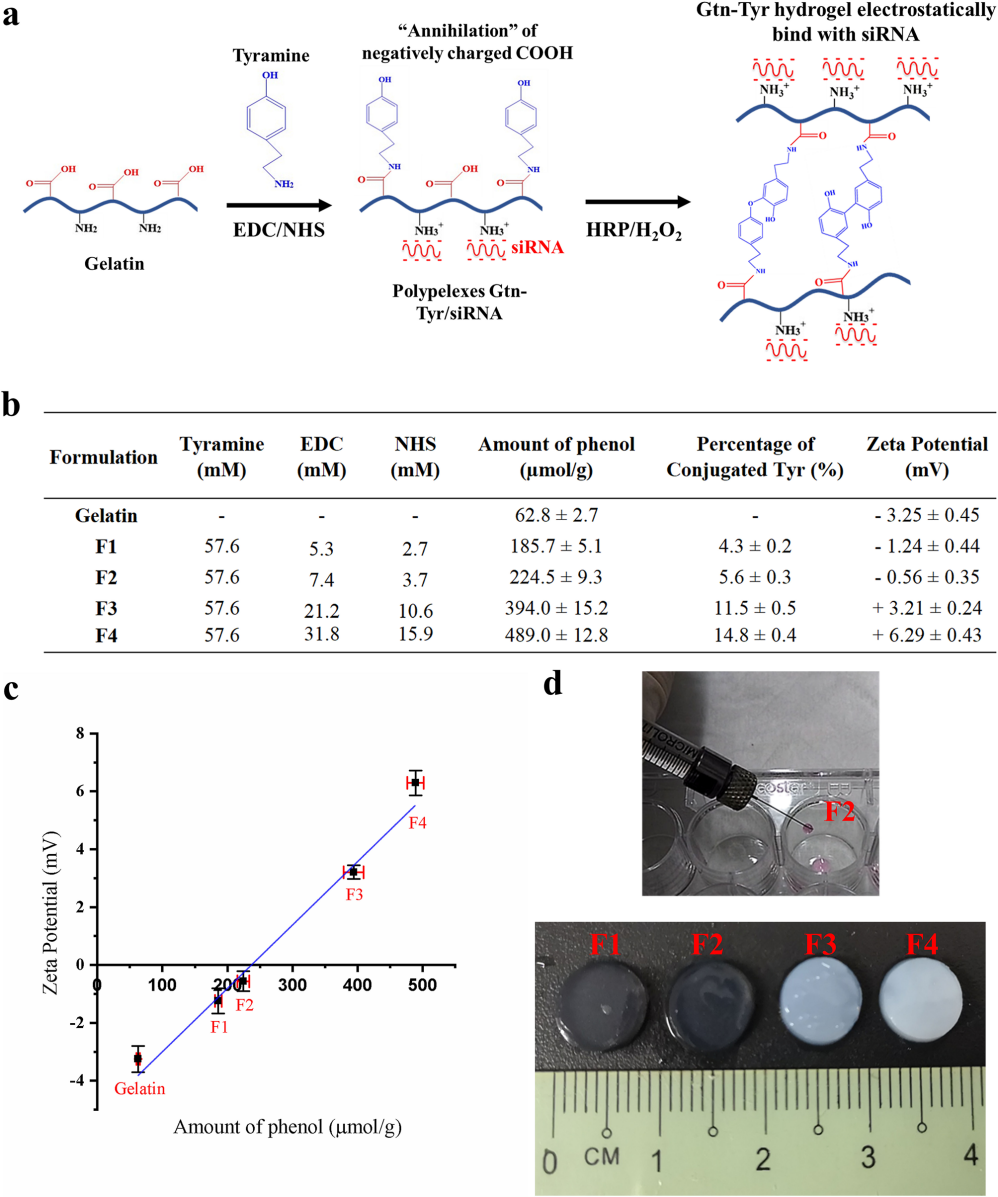
(**a**) A scheme showing a two-step hydrogel synthesis process involving the introduction of tyramine for positive-charge tuning and crosslinking bifunctionality, follow by peroxidase-mediated phenol coupling for hydrogel-siRNA formation. (**b**) A table showing various formulations used for the synthesis of gelatin-tyramine (Gtn-Tyr) precursors, resulting in an increasing percentage of conjugated tyramine and zeta potential. (**c**) A graph showing a linear relationship between the increasing amount of conjugated Tyr with phenol group and zeta potential. (**d**) Gtn-Tyr precursor displaying ease of injectability through a 32G Hamilton syringe, with the hydrogel formed displaying increasing opacity with increasing zeta potential.

The current work reports the synthesis of a positive-charge tuned gelatin hydrogel and its application as an efficient delivery platform for siSPARC in cellular and animal models. We develop a simple two-step synthesis of a gelatin hydrogel delivery system that enables tunability of its surface charge properties while simultaneously introduces the crosslinking sites on the polymer’s backbone, simplifying the synthesis process while preventing siRNA lost during the process (Fig. 1a). In our synthesis strategy, gelatin, a heterobifunctional biocompatible which consists of both positively charged (e.g. amino) and negatively charged (e.g. carboxyl) functional groups, is used as the chemical building block. Tyramine (Tyr) is conjugated onto gelatin to function as both charge tuning and crosslinking moiety. Tyramine molecules have two functional groups, which are amino and phenol groups. The charged amino groups on Tyr is used to ‘annihilate’ the free negatively charged carboxyl groups found on the gelatin to create a net positively charged environment for the gelatin, while the non-charged phenol group is utilized as a crosslinking site for gelatin hydrogel formation. As the net “free” positively charged amino groups on the gelatin are not involved in the crosslinking process, the electrostatic properties and charge magnitude can be maintained even after the formation of hydrogel’s network. By exploiting gelatin’s naturally endocytosed and tunable surface charge properties, the positive-charge tuned gelatin hydrogel is systematically investigated in vitro for SPARC gene silencing and down-regulation by varying its mechanical and surface charge properties. We further demonstrate the positive-charge tuned gelatin hydrogel as an injectable for effective siSPARC delivery in post-GFS anti-fibrotic treatment in a rabbit model.

## Materials and methods

### Synthesis of Gtn-Tyr precursor

Gelatin was conjugated with small phenol molecule, tyramine, using the common carbodiimide crosslinking reaction and previously established procedures by Wang et al.^37^. Briefly, 2 w/v% of gelatin (Wako Pure Chemical Industries, Ltd., Japan) was dissolved in DI water by heating the solution at 60 °C. 57.6 mM Tyramine chloride (Tyr.Cl; Sigma-Aldrich, US) was then added to this solution. 5.3–31.8 mM N-(3-Dimethylaminopropyl)-N′-ethylcarbodiimide hydrochloride (EDC.HCl; Sigma-Aldrich, US) and 2.7–15.9 mM N-Hydroxysuccinimide (NHS; Sigma-Aldrich, US) were added into the mixture to initiate the conjugation reaction. The reaction was allowed to proceed overnight at pH 4.7. After the reaction, the mixture was purified, dialyzed and finally freeze-dried to obtain the hydrogel precursor. The precursor was then scanned with absorbance at 275 nm (phenol group) using UV–Vis spectrometry to quantify the amount of tyramine conjugated onto the gelatin backbone. The absorbance of the precursor was compared with the absorbance of the known amount of Tyr. Each sample was tested three times, and the average of 3 different samples synthesized using the same condition was calculated.

### Zeta potential measurement

The zeta potential of Gtn-Tyr precursors with different conjugation degrees was measured using the Malvern Zetasizer (Malvern Panalytical Ltd, UK). The freeze-dried precursors were dissolved in DI water with a concentration of 5 w/v%. The zeta potential was measured in the DTS1070 disposable folded capillary cells at the temperature of 25 °C. The instrument was calibrated using latex with known zeta potential. Each sample was tested three times and the average of 3 different samples synthesized using the same condition was calculated.

### Fabrication of Gtn-Tyr hydrogel

The freeze-dried Gtn-Tyr precursor was dissolved in 1 × PBS to form 5 w/v% precursor solution. The precursor solution was then crosslinked using different amount of horseradish peroxidase (HRP, Wako Pure Chemical Industries, Japan) and 3 mM hydrogen peroxide (H_2_O_2_, ACROS Organics, US).to form Gtn-Tyr hydrogel.

### siRNA

A 21-base double-stranded small interfering RNA for SPARC (siSPARC: 5′-AACAAGACCUUCGACUCUUCC-3′) was used for in vitro and in vivo SPARC knockdown studies. A non-silencing scrambled control (siScramble: 5′-GCUCACAGCUCAAUCCUAAUC-3′) was also used. Fluorescence tagged SPARC, FAM-SPARC, was used for release studies. The siRNAs were synthesized and purified by Bioneer (Korea).

### Cell internalization study

Human dermal fibroblast (HDFs) were seeded with a cell density of 5 × 10^4^ cells/well on 24 well plate and cultured using Dulbecco's Modified Eagle Medium (DMEM) high glucose supplemented with 10% fetal bovine serum (FBS, Thermo Fisher Scientific, US) and 1% Antibiotic–Antimycotic (ABAM, Thermo Fisher Scientific, US). Gtn-Tyr with the highest positive surface charge (ZP =  + 6.29 ± 0.43 mV) was used in this proof-of-concept study to ensure electrostatic interaction with siSPARC. Once HDFs reached ~ 80% confluency, the culture medium was removed and replaced with culture medium containing FAM-siSPARC or polyplexes of FAM-siSPARC/Gtn-Tyr. The polyplexes of FAM-siSPARC/Gtn-Tyr was formed through mixing 2 nmol FAM-siSPARC with 5 w/v % Gtn-Tyr solution, vortexed thoroughly to ensure the siSPARC was distributed homogeneously, and allowed them to interact for 15 min. The polyplexes was then added into the culture medium to obtain a final siSPARC concentration of 4 nmol/ml. HDFs were incubated with the FAM-siSPARC or polyplexes of FAM-siSPARC/Gtn-Tyr for 24 h under standard culture conditions. After the treatment, HDFs were stained with Hoechst33342 (Life Technologies, US) according to manufacturer protocol. HDFs were then imaged using a ZEISS Axio Observer Z1 fluorescence microscope (ZEISS, Germany). All imaging settings were kept constant for any comparisons between experimental conditions.

### FAM-SPARC release profile

5 w/v% Gtn-Tyr solution was prepared by dissolving freeze-dried precursor into 1 × PBS. 400 pmol FAM-SPARC was then loaded into the 0.1 ml Gtn-Tyr solution. The precursor mixture was vortexed thoroughly to ensure the siRNA was distributed homogeneously inside the gel before the crosslinking process. HRP and H_2_O_2_ with a final concentration of 0.12 unit/ml and 3 mM respectively were added to the mixture and then cast into 96 well plates. The sample was allowed to set for 0.5 h before topping up with 0.1 ml of 1 × PBS as a release buffer solution. As no multiple steps or washing is involved during the siSPARC encapsulation, 100% siSPARC loading efficiency was expected using our synthesis and fabrication strategy. At a specific time, the release buffer solution was collected and replenished with fresh solution. The collected release buffer solution was then scanned using an excitation wavelength of 492 nm and an emission wavelength of 517 nm (SpectraMax M2 microplate readers, Molecular Devices, US). The fluorescence intensity was compared with the fluorescence intensity of a known amount of FAM-SPARC.

### In vitro SPARC silencing of C57Bl6/J mouse tenon fibroblasts (MTFs) using the electrostatic tunable Gtn-Tyr hydrogel

In vitro SPARC silencing and characterization were performed using a previously established method with slight modification^23^. Briefly, freeze-dried Gtn-Tyr was dissolved with DMEM high glucose without FBS (5 w/v%) and loaded with 4 nmol/ml siSPARC. The hydrogel precursor with siSPARC was sterilized using 0.22 µm syringe filter. Next, 0.5 ml Gtn-Tyr hydrogel containing siSPARC was fabricated by adding HRP and H_2_O_2_ with a final concentration of 0.15 units/ml and 3 mM respectively. The mixture was then cast onto a 12 well plate. C57Bl6/J MTFs were seeded on top of the hydrogel with a density of 3 × 10^4^ cells per well. The sample was topped up with 0.5 ml culture medium and incubated at 37 °C and 5% CO_2_. At day 2 and 7, total RNA was recovered with Trizol Reagent (Invitrogen Corp., US) according to the manufacturer's recommendations. First-strand cDNA was synthesized with 500 ng total RNA extract and 1 µl of 50 ng/µl random hexamer primer (Invitrogen Corp., US) with Superscript III reverse transcriptase (Invitrogen Corp., US) according to the manufacturer's instructions. Quantitative real-time PCR (qPCR) was performed in a total volume of 10 µl in 384-well microtiter plates. Each reaction consisted of 1 µl of the first-strand reaction product, 0.5 µl each of upstream and downstream primers (10 µM each), 4 µl of Power SYBR Green PCR Master Mix (Applied Biosystems, US), and 4 ul of DNase-RNase-free distilled water (Sigma-Aldrich, US). Amplification and analysis of cDNA fragments were carried out by use of the Roche LightCycler 480 System (Roche Diagnostics Corp, US). All PCR reactions were performed in triplicate. All mRNA levels were measured as CT threshold levels and were normalized with the corresponding 18S CT values (housekeeping gene). Values are expressed as fold increase over the corresponding values for untreated WT control by the 2 − ΔΔCT method.

### Western blot

After treated with Gtn-Tyr hydrogel containing 4 nmol/ml siSPARC or siScramble for 7 days, C57Bl6/J MTFs was extracted and lysed using a solution containing 20 mM Tris buffer (pH 7.4), 150 mM NaCl, 1 mM EDTA, 0.5% Triton X-100, 2 mM MgCl_2_, 1 mM dithiothreitol, and 1 × Complete Protease Inhibitors (Roche Diagnostics GmbH, Germany). SDS–polyacrylamide gel electrophoresis and immunoblotting were performed using the antibodies against SPARC, β-tubulin, GAPDH (Santa Cruz Biotechnology, Inc., US), collagen I (Novus Biologicals, US) and horseradish peroxidase (HRP)–conjugated secondary antibodies (Jackson Immunoresearch Laboratories, Inc., US). Densitometric quantitation was performed according to a previously established protocol^38^, and potential errors in loading were corrected to levels of GAPDH (housekeeping protein).

### Annexin V assay by flow cytometry

The cytotoxicity of Gtn-Tyr hydrogel loaded with 4 nmol/ml siSPARC was assessed through C57Bl6/J MTF’s apoptosis studies using the Guava Nexin Reagent (Guava Technologies, Hayward, CA). The nexin assay is based on the measurement of annexin V that binds to phosphatidylserine, which will translocate from the inner to the outer surface of the cell membrane during apoptosis. After treated with 4 nmol/ml siSPARC containing Gtn-Tyr hydrogel for 7 days, C57Bl6/J MTFs were trypsinized and processed according to the manufacture’s protocol. 3000 cells from each sample were analyzed. Cell populations were quantified using the Guava EasyCyte Plus flow cytometry system (Guava Technologies, Hayward, CA), and the data were analyzed using the Guava Nexin software (Guava Technologies, Hayward, CA).

### Surgical procedure

All experiments with animals were approved by the Institutional Animal Care and Use Committee (IACUC) and treated following the Association for Research in Vision and Ophthalmology (ARVO) Statement on the Use of Animals in Ophthalmic and Vision Research. Fifteen New Zealand White rabbits (2–2.4 kg, 12–14 weeks old; SEMC, Duke-NUS, NCCS Animal Facility, Sembawang, Singapore) were acclimatized for 7 days before the experiment commenced. Surgical procedure was performed according to a previously established method^39^. Briefly, the animals were anaesthetized with a combination of ketamine (Ketaset; Fort Dodge Animal Health, UK) and medetomidine HCl (Domitor; Pfizer Animal Health, UK). A fornix based conjunctival flap was raised, and blunt dissection of the subconjunctival space was performed of approximately 3 mm along the limbus and 5 mm posteriorly. A 24-gauge, 25-mm intravenous cannula (Venflon 2; Beckton Dickinson, UK) (which is the smallest cannula available for this modified tube surgery) was used to create a sclerotomy, starting 2.5 mm behind the limbus, passing into clear cornea before entry into the anterior chamber. The needle was then withdrawn and removed as the cannula was advanced into the mid-pupillary area. The cannula was then trimmed and bevelled at its scleral end to protrude 1 mm from the insertion point. A 10–0 nylon suture (B/V 100–4; Ethicon, US) fixed the tube to the scleral surface. Closure of the conjunctival incision was performed using 10-0nylon via purse-string sutures and where necessary a mattress suture. One drop each of guttae chloramphenicol and Betnesol-N (Glaxo Wellcome, UK) ointment was instilled at the end of surgery. Only the left eye was operated on, and the surgical procedure was performed at the same site superiorly in each animal.

### In vivo treatment regimen

Five rabbits each were randomly allocated to one of three treatment groups: (1) subconjunctival injection of 0.1 mL Gtn-Tyr hydrogel loaded with 2 nmol/ml siSPARC, (2) subconjunctival injection of 0.1 mL Gtn-Tyr hydrogel loaded with 2 nmol/ml siScramble or (3) 0.2 mg/ml MMC application for 1 min to the subconjunctival space during surgery. The hydrogel precursor with siSPARC was sterilized using 0.22 µm syringe filter before injection. Groups receiving the subconjunctival hydrogel injection had it administered immediately after surgery and on Days 3, 7 and 9 post-operatively. Topical antibiotic and steroid drops were administered daily for 2 weeks post-operatively. The animals were sacrificed on day 30. Only the left eye was enucleated and histologically analysed.

### Examination and clinical evaluation

Post-operative observations were performed at weekly intervals until sacrifice, according to a previously established method^39^. A single, masked independent investigator objectively graded each bleb for survival and vascularity based on slit-lamp examination and photography. The primary outcome metric was bleb histology and bleb survival, which was defined as the presence of an elevated subconjunctival fluid pocket at the surgical site. Slit-lamp microscopy was performed using Righton LED slit lamp MW50D (Right Mfg Co Ltd, Japan). In vivo confocal microscopic examinations of the operated and treated conjunctiva were performed using Hrt3 microscope (Heidelberg Engineering, Heidelberg, Germany). Optical coherence tomography angiography of the bleb vasculature was captured Optovue AngioVue (Optovue, Inc., US).

### Histological evaluation

Both eyes were enucleated. The upper lid was removed together with the whole eye to preserve the bleb and superior conjunctiva. Histological evaluation of operated conjunctival cryosections by H&E, Picro-Sirius Red and Masson’s Trichrome staining was performed. Qualitative clinical and histological evaluation was vital in the study. Survival analysis could not be performed by the Kaplan–Meier log-rank test as is usually done because hydrogel component did not dissipate for at least 2 weeks after injection, confounding findings which could not be impartially compared to the MMC group until the final week.

### Statistical analysis

All data are expressed in mean ± standard deviation with a replicate of n = 3 unless otherwise specified. The differences between the values were assessed using one-way ANOVA, where *P* < 0.05 was considered statistically significant. Differences are labelled * for *P* ≤ 0.05, ** for *P* ≤ 0.01, *** for *P* ≤ 0.001 and **** for *P* ≤ 0.0001.

## Results and discussion

### Gtn-Tyr hydrogel with tunable surface charge properties

Gelatin was chosen as the polymer candidate for the current surface charge tunable hydrogel fabrication, considering its established biocompatibility^27^ and naturally-endocytosed property by fibroblasts^40^. Fibroblasts are mainly responsible for the production and distribution of extracellular matrix (ECM) proteins such as collagen, and their excessive proliferation is associated with fibrosis^41^. As the current work is concerned with the protection and delivery of negatively charged siRNA, the free carboxyl groups on the gelatin were “annihilated” by the amino groups on tyramine molecules, creating a net positively charged environment contributed by the remaining free amino groups on the gelatin (Fig. 1a). The conjugation was done through carbodiimide crosslinking reaction between the amino group of tyramine and the carboxyl group of gelatin using EDC through the formation of amide bonds^42^, thus resulting in the overall reduction of the number of negatively charged carboxyl groups on the gelatin.

The current hydrogel fabrication strategy has a unique feature of employing only one moiety, i.e. tyramine, to endow charge and crosslinking properties simultaneously. However, the surface charge and crosslinking density of the hydrogel system can still be adjusted independently. While the charge is controlled by the amount of “annihilation” by Tyr on the gelatin backbone, the crosslinking reaction is independently controlled by the amount radicals generated for phenol coupling from the enzymatic reactions between HRP and H_2_O_2_^43,44^.

We controlled the percentage of tyramine conjugated onto the gelatin and hence its overall surface charge property through adjusting the concentration of EDC and NHS during the synthesis process, with an excess of tyramine used. The percentage of tyramine conjugated to gelatin was varied between 4 and 15% (Fig. 1b). Unmodified gelatin had a zeta potential of − 3.25 ± 0.45 mV. With an increasing amount of tyramine conjugated, resulting in increasing amount of amino groups “annihilating” the carboxyl groups on the gelatin, the zeta potential of gelatin showed a linear increase towards a more positive value up to + 6.29 ± 0.43 mV (Fig. 1c). It is noteworthy that the Gtn-Tyr precursor solutions, regardless of zeta potential values, exhibit superior “water-like” injectable property and easily passed through a small 32-gauge needle before gelation (Fig. 1d).

In the crosslinking reaction, the HRP was varied at 0.12 – 0.15 units/ml while an excess amount of H_2_O_2_ at 3 mM was used. This is to ensure a pseudo-first-order reaction condition such that the amount of conjugated Tyr with phenol group remained as the only variable. Also, we have shown that the stability of siSPARC was not affected by the treatment of 3 mM H_2_O_2_ (Fig. S1). It follows that an increasing conjugated Tyr amount (corresponded to an increasing amount of phenol groups which function as crosslinkers) will generate a hydrogel of increasing positive charge and crosslinking density. Upon gelation, the hydrogel formed displayed increasing opacity with increasing zeta potential (Fig. 1d). We attribute this to the increasing positive charge environment between different gelatin chains resulting in phase separation during the hydrogel fabrication process^45–47^. Rheology analysis (Fig. S2) showed an optimal storage modulus for sample F2 (storage modulus 3.3 ± 0.5 kPa) and subsequently decreasing storage modulus or stiffness with increasing zeta potential (sample F3 and F4 with storage moduli 0.8 ± 0.1 and 0.2 ± 0.1 kPa respectively), suggesting that phase separation has occurred as a result of the positive-charge tuned gelatin chains repelling each other. Hydrogel samples were subjected to collagenase digestion to investigate the degradation rate of the hydrogel when the amount of conjugated tyramine increased. Hydrogel degradation studies (Fig. S3) performed on the various hydrogel formed showed decreasing degradation rate with increasing zeta potential, attributed to increasing crosslinking density with an increasing amount of conjugated tyramine. Besides “annihilating” the carboxyl groups, Tyr also serves as the crosslinking moiety for the formation of Gtn-Tyr hydrogel. Therefore, increasing the amount of Tyr conjugation on the gelatin backbone resulted in an increase in zeta potential as a greater amount of conjugated Tyr is directly correlated to a higher crosslinking density.

From a mechanotransduction perspective, matrix stiffness plays a critical role in wound healing and fibrosis, where stiffer matrix (> 5 kPa) has been shown to encourage fibroblast differentiation and progression^23,48–50^. The current hydrogel fabricated is considered soft (< 3.5 kPa for all samples) thus suitable as a therapeutic delivery platform for wound healing and fibrosis treatment. Taken together, we have fabricated a type of gelatin hydrogel with tunable surface charge property via a simple two-step process. A single bifunctional biomolecule, tyramine, is used for crosslinking and charge tuning, giving rise to a biocompatible and scalable hydrogel system displaying increasing zeta potential with increasing conjugated tyramine amount and crosslinking density (from sample F1 to F4), decreasing hydrogel degradation rate, and an optimal storage modulus for hydrogel sample F2. It is anticipated that these properties will collectively interact with the negatively charged siRNAs, affecting their release kinetics, protection from RNAses degradation and cellular internalization. These physiochemical interactions with the encapsulated siRNAs will be studied using cellular and animal models in the proceeding sections with a goal of optimizing the gene silencing efficiency of siSPARC for anti-fibrosis treatment.

### siRNA encapsulated in Gtn-Tyr hydrogel: In vitro evaluation of cell internalization, siSPARC release, silencing and cytotoxicity.

**Figure 2:**
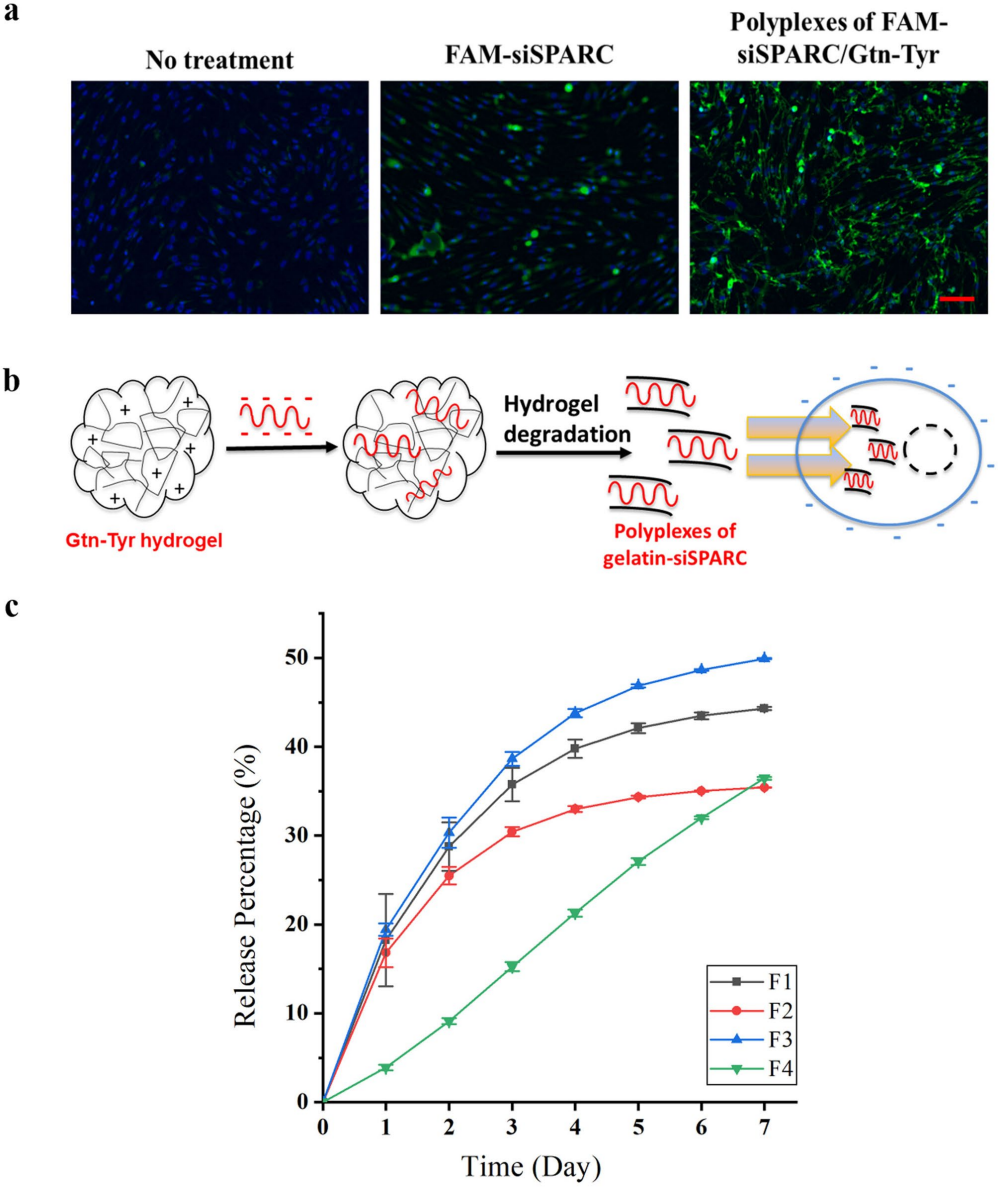
(**a**) Representative images of HDFs without treatment, incubated with 4 nmol/ml naked FAM-siSPARC, and 4 nmol/ml FAM-siSPARC /Gtn-Tyr (left to right). FAM-siSPARC is indicated in green. Cell nuclei stained with Hoechst 33,342 is indicated in blue (Scale bar represents 100 μm). (**b**) Formation of polyplexes between siSPARC and positive-charge tuned Gtn-Tyr during hydrogel degradation in the presence of collagenase increased cell internalization. (**c**) siSPARC release profile from the Gtn-Tyr hydrogels with different Tyr amount (affecting surface charge and crosslinking density).

A safe and efficient siRNA protection and delivery to the cellular environment remains the critical bottleneck of siRNA therapeutics for clinical application. Herein, we demonstrated the ability of Gtn-Tyr in protecting and enhancing siSPARC delivery into the cellular environment using human dermal fibroblasts (HDFs) (Fig. 2a). HDFs are involved in wound healing and an increase in SPARC production in HDFs was linked to fibrosis and scarring^51,52^. Comparing different treatment groups (Fig. 2a), HDFs treated with polyplexes of FAM-siSPARC/Gtn-Tyr (right) showed a significantly higher green fluorescence intensity as compared to gelatin-absent FAM-siSPARC (middle). This observation supports that the positive-charge tuned gelatin, when electrostatically interacted with negative-charge siSPARC, was endocytosed by fibroblasts into the cytoplasm. We have further verified the electrostatic interaction between the negatively charged siRNA and positive-charge tuned Gtn-Tyr by showing a significant reduction (*P* < 0.0001) in overall zeta potential after the interaction between these two components (Fig. S4). The current cell internalization study demonstrated the importance of using the positive-charge tuned Gtn-Tyr hydrogel to form polyplexes with siSPARC to enhance the protection and delivery of SPARC into cellular environment.

We proposed a mechanism to describe the enhancement of siSPARC cell internalization through formation of polyplexes with the Gtn-Tyr hydrogel (Fig. 2b). Polyplexes were first formed between siSPARC and Gtn-Tyr during hydrogel formation. During hydrogel degradation, the siSPARC electrostatically bound to the positive-charge tuned Gtn-Tyr is subsequently internalized by the cells through a clathrin-mediated endocytotic pathway^31^. The degraded products of the Gtn-Tyr hydrogel, regardless of their different formulations, were found to have an average size below the upper size limit (200 nm) of the clathrin-mediated endocytosis mechanism^53^ (Fig. S5a). The mechanism of the endosomal escape of the siSPARC-hydrogel is proposed to be due to the “proton sponge” effect of positive-charge tuned Gtn-Tyr^54^. The positive-charge tuned Gtn-Tyr acts as a proton barrier to prevent acidification of siSPARC within the endo-lysosomal compartments. Finally, endo-lysosomes lysis due to the osmotic imbalance in the presence of the cationic Gtn-Tyr resulted in the release the polyplexes within the cytoplasm.

Next, siSPARC release studies were performed to investigate the effect of increasing amount of conjugated Tyr, which resulted in increasing zeta potential and thus electrostatic interaction between the gelatin backbone and siSPARC. The release studies (Fig. 2c) confirm the current hydrogel’s ability to provide a sustained release of siSPARC, albeit with different release characteristics depending on the amount of tyramine conjugated in the hydrogel system. When hydrogel F1 and F2 (zeta potential values at − 1.24 ± 0.44 mV to − 0.56 ± 0.35 mV respectively) are compared, a decrease in the release rate was observed, attributed to the increase in both crosslinking density and surface charge. A higher crosslinking density reduces biomolecule/drug release while a more positive surface charge promotes stronger electrostatic interactions between the gelatin and siSPARC, reducing siSPARC release. However, further increase in zeta potential from − 0.56 ± 0.35 mV (F2) to + 3.21 ± 0.24 mV (F3) (corresponding increase in crosslinking density) showed an increase in the overall release rate. This is due to the weaker hydrogel structure attributed to phase separation. Finally, sample F4 of the highest zeta potential of + 6.29 ± 0.43 mV showed the slowest initial release rate, probably due to a high positive surface charge being the dominant factor in binding the siSPARC. The electrostatic interaction between siRNA and F4 is expected to be the strongest despite its relatively weak structure, contributing to the slowest initial release of siRNA. At day 7, its release surpassed that of sample F2 most likely due to enhanced siRNA release within the phase-separated hydrogel network. However, the siRNA release rate from Gtn-Tyr hydrogel, regardless of the amount of surface charge, is predicted to be higher in vivo due to the natural presence of collagenases (matrix metallopeptidases) in living tissues.

**Figure 3:**
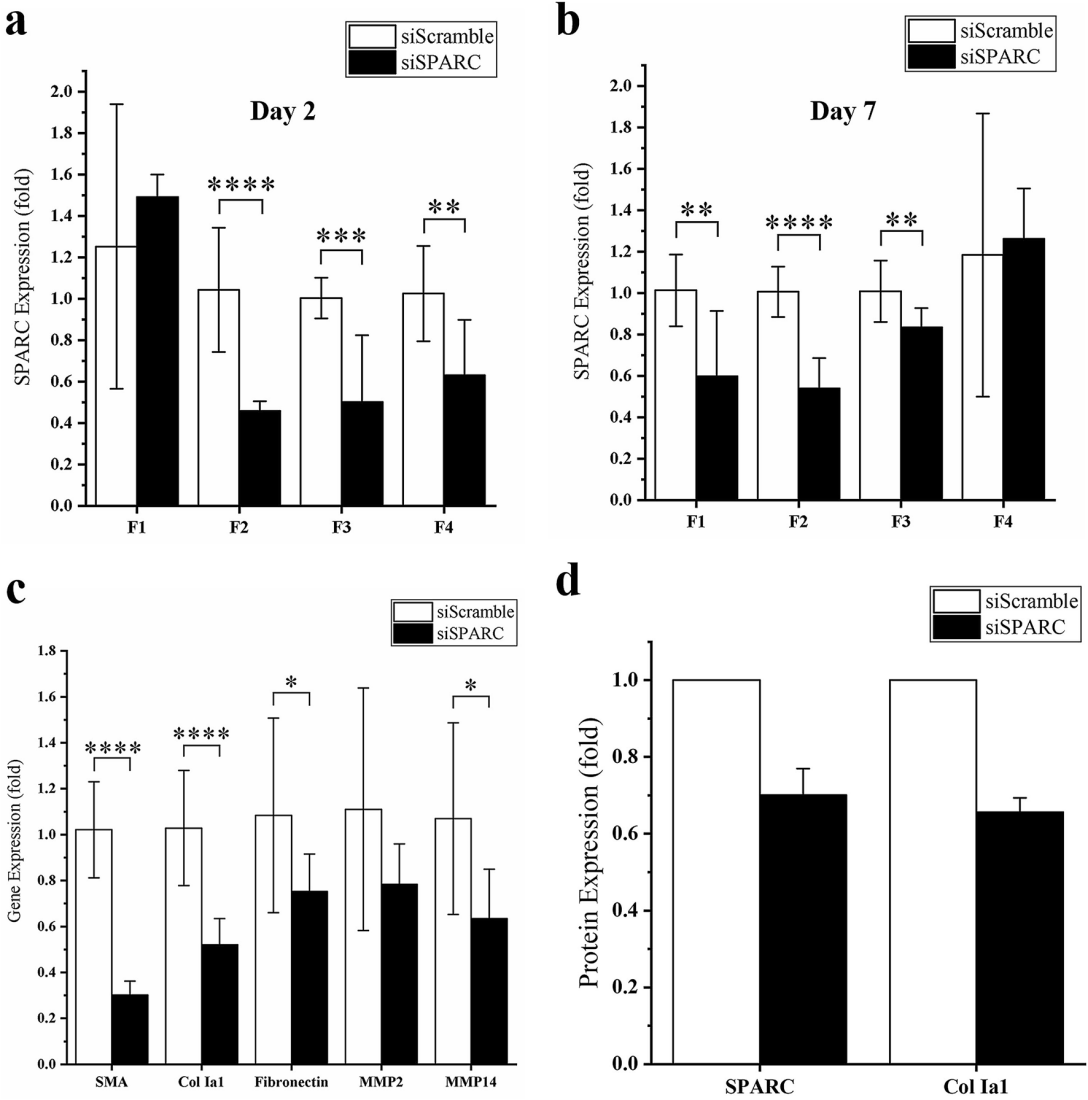
SPARC expression of C57Bl6/J MTFs at day (a) 2 and (**b**) 7 when treated with siSPARC-loaded Gtn-Tyr hydrogel with different surface charges. (**c**) Gene expression of SMA, Col 1a1, fibronectin, MMP2 and MMP4; and (**d**) SPARC and Col 1a1 protein expression of C57 MTFs after being treated with optimal hydrogel formulation (F2) at day 7. * denote *P* < 0.05, ** denote *P* < 0.01, *** denote *P* < 0.001 and **** denote *P* < 0.0001.

siSPARC release characteristics from the current gelatin hydrogel system were affected by a combination of physiochemical properties of the hydrogel, including surface charge, crosslinking density, and mechanical strength. We study how these properties can be integrated to optimize gelatin hydrogel-siSPARC interactions to enhance the effectiveness of SPARC gene knockdown in F1-F4 Gtn-Tyr hydrogel samples using MTFs. In an in vitro siSPARC dosage optimization study, 4 nmol/ml siSPARC was found to be an effective dose as compared to 1 or 2 nmol/ml (Fig. S5) and used to compare the effectiveness of F1-F4 hydrogels in gene knockdown studies. MTFs treated with negatively charged hydrogel sample (F1) did not show any gene knockdown on day 2 (Fig. 3a). This might be due to an absence of electrostatic interaction between negatively charged Gtn-Tyr and siSPARC and therefore, an absence of gelatin-assisted protection and cell internalization. However, shifting the net surface charge to more positive (F2-F4 samples), as compared to raw gelatin (zeta potential =  − 3.24 ± 0.45 mV) was shown to result in a significant SPARC knockdown at day 2. Thus, the in vitro SPARC knockdown results in Fig. 3a reinforced the proposed mechanism of cell internalization (Fig. 2b) through polyplexes formed between the positive-charge tuned hydrogel and siSPARC. Meanwhile, the F4 Gtn-Tyr hydrogel with the highest positive surface charge showed the lowest knockdown effect on day 2 amongst the samples with more positive net surface charge, attributed to the strongest electrostatic interaction, and hence the slower release rate. MTFs exposed to F2 siSPARC-loaded Gtn-Tyr hydrogel demonstrated highest SPARC mRNA knockdown after treatment for 2 days (~ 54%, *P* < 0.0001; Fig. 3a) and 7 days (~ 46%, *P* ≤ 0.0001; Fig. 3b). To further confirm if F2 affect downstream scarring gene expression, we determined that mRNA expression of SMA, Col 1a1, fibronectin, MMP2, and MMP14 treated with F2 for 7 days were downregulated by 72% (*P* < 0.0001), 51% (*P* < 0.0001), 33% (*P* < 0.05), 33% and 44% (*P* < 0.05) respectively (Fig. 3c). We also verified that both SPARC and Col Ia1 protein expression in F2-treated cells was reduced by more than 30% at the protein level (Fig. 3d). Representative western blot image is shown in Fig. S7 and S8 in supporting information. Furthermore, the degradation products of F2 hydrogel samples incorporated with siSPARC were determined to have an average size of about 100 nm, well below the clathrin-mediated endocytosis upper size limit of 200 nm (Fig. S5b)^53^.

**Figure 4:**
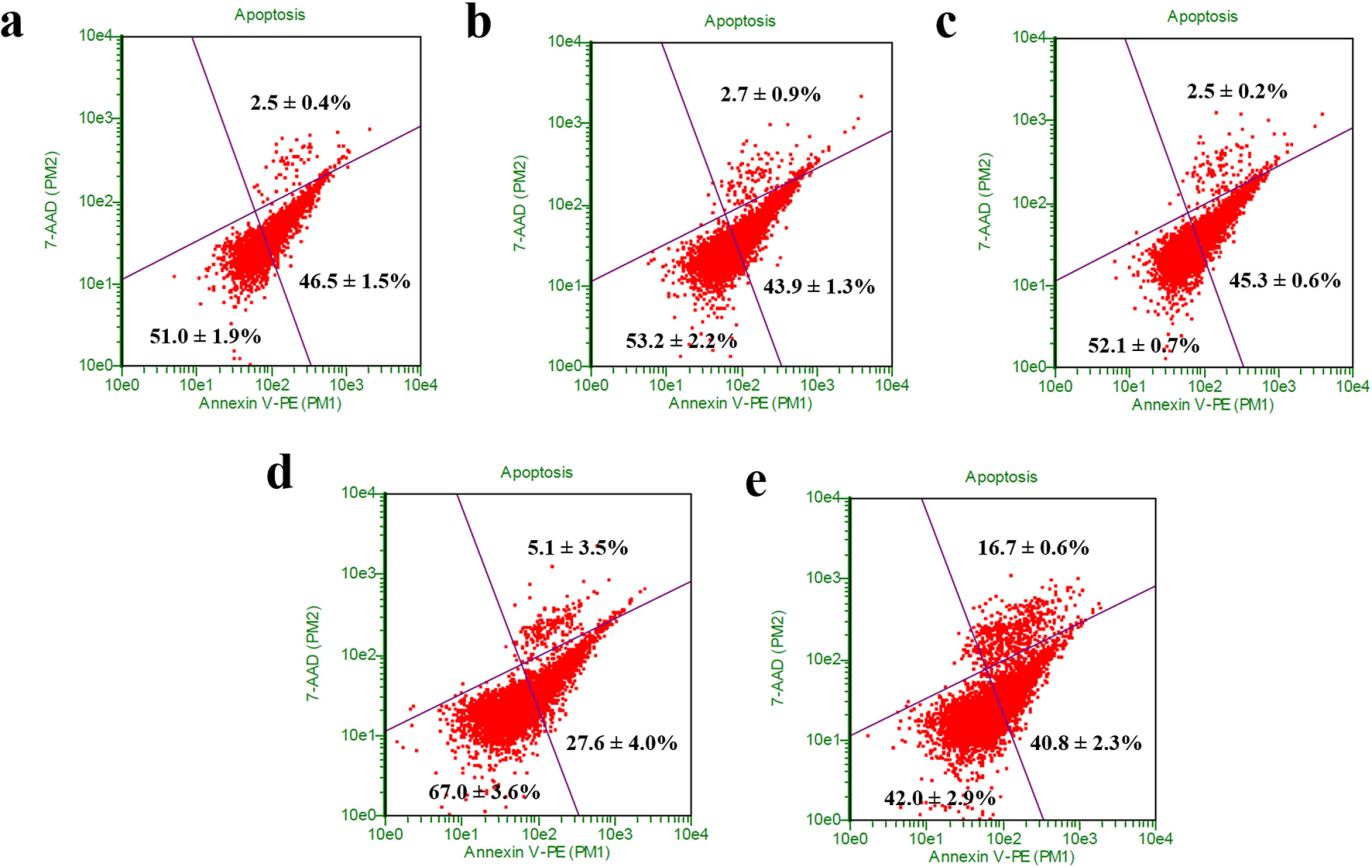
Cytotoxicity studies of (**a**) F2 Gtn-Tyr hydrogel-only, (**b**) siScramble loaded Gtn-Tyr hydrogel and (**c**) siSPARC loaded Gtn-Tyr hydrogel as compared to controls (d) untreated cells and (**e**) apoptosis-induced cells. After treated for 7 days, C57Bl6/J MTFs were stained by Guava Nexin reagent containing 7-AAD/Annexin-V-PE and analysed by flow cytometry. In the four windows of each plot, the lower-left indicates healthy cells, the lower right indicates early apoptotic cells, and the upper right indicates late-phase apoptotic cells or necrotic cells.

We also evaluated the cytotoxic effects of F2 via apoptosis studies. As shown in Fig. 4, the MTFs treated with F2 hydrogel only, siScramble-loaded F2 hydrogel and siSPARC-loaded F2 hydrogel showed a significantly increase (*P* < 0.05) in the percentage of the early-stage apoptotic cell as compared to the untreated cell at day 7. The untreated cell consisted of 27.6 ± 4.0% of early-stage apoptotic cell. A significant increase to 46.5 ± 1.5% (*P* < 0.01) , 43.9 ± 1.4% (*P* < 0.01) , and 45.3 ± 0.5% (*P* < 0.01) was observed when the cells were treated with Gtn-Tyr hydrogel only, siScramble-loaded F2 hydrogel and siSPARC-loaded F2 hydrogel respectively. However, there is no significant increase and differences in the percentage of the early-stage apoptotic cell among the hydrogel groups. Therefore, the increase in the early-stage apoptotic cell might be due to the F2 hydrogel alone and not from the siRNA. When comparing the percentage of the late-stage apoptotic cell, there is no difference between the untreated cell (5.1 ± 3.5%) with F2 hydrogel without siRNA (2.5 ± 0.4%), with siScramble (2.7 ± 0.9%) or siSPARC (2.5 ± 0.2%). All the sample groups were significantly lower in percentage (*P* < 0.05) as compared to induced apoptosis group (16.7 ± 0.6%). To further investigate cytocompatibility, F2 hydrogel was subjected to cell proliferation and viability studies. The cell proliferation result (Fig. S9a) showed that MTFs were able to attach and proliferate on the surface of F2 hydrogel. Besides, cell viability result (Fig. S9b) showed that MTFs were viable on the hydrogel’s surface at day 1, 3 and 7 without excessive dead cells. Overall, the F2 hydrogel did not cause a toxic effect to the cells and allowed the cell attachment and proliferation. As a contrast, F2 hydrogel induced ~ 18% more early apoptosis, which might be an advantage and strategy for anti-scarring through reducing and “controlling” the surrounding number of fibroblasts^55–57^. However, this effect needs to be further investigated to understand the source and mechanism of causing early apoptosis fully.

### In vivo evaluation using GFS rabbit model

**Figure 5:**
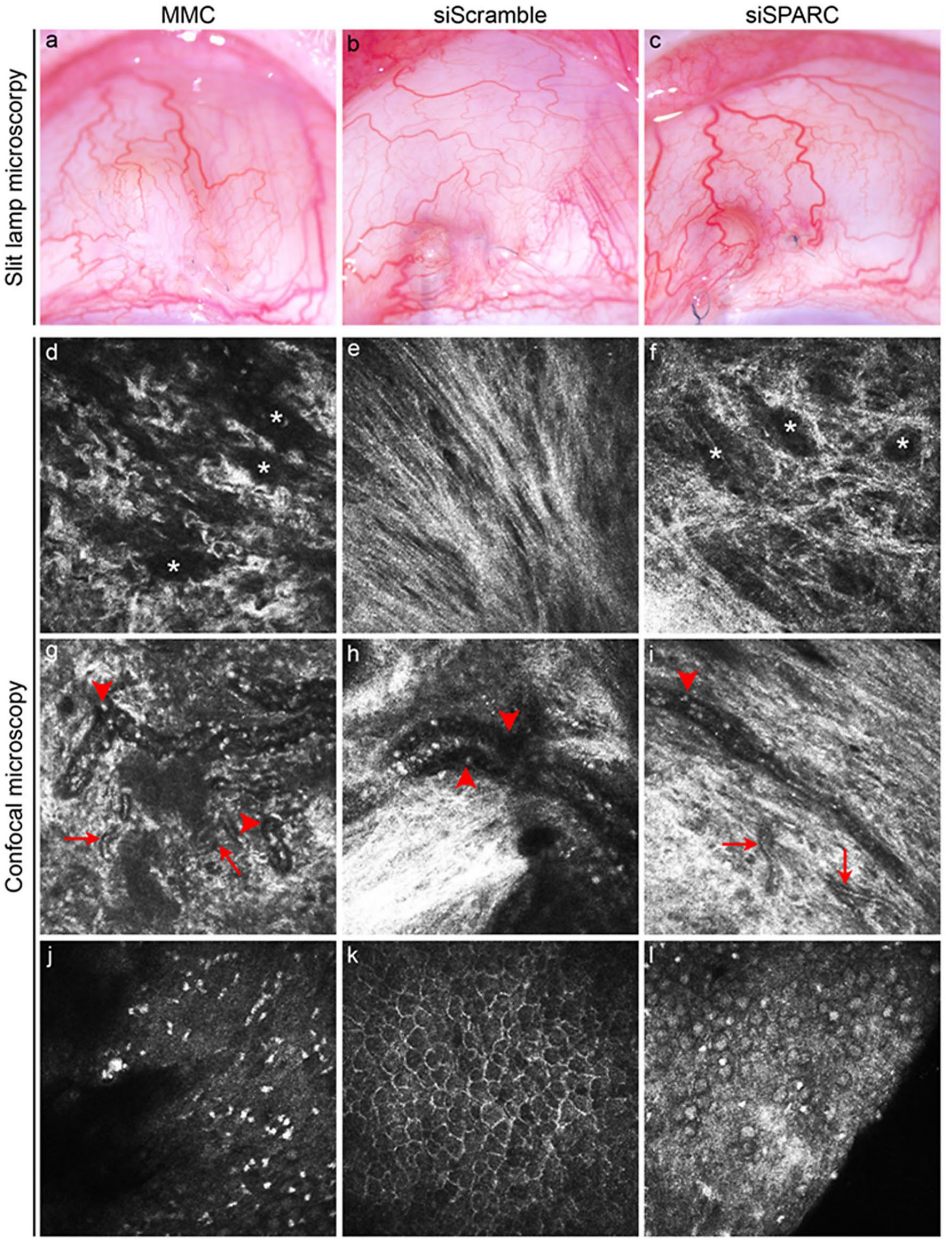
Live imaging of operated tissues in a rabbit model of glaucoma filtration surgery with insertion of a 24-gauge cannula**.** (**a**–**c**) Slit-lamp microscopy of the week 4 postoperative tissues treated with MMC, or hydrogel incorporated with scrambled or SPARC siRNAs. While blebs were no longer visible in all the MMC or siScramble-hydrogel treated tissues, 4 of 5 siSPARC-hydrogel treated tissues maintained visible blebs at week 4. (**d**–**l**) In vivo confocal images of the week 4 operated area. *, microcysts; red arrowheads, large vasculature; red arrows, fine vasculature.

The F2 Gtn-Tyr hydrogel system, which was found to have optimal positive-charge tuned environment and release characteristics, was evaluated for its effectiveness in enhancing anti-scarring effect in vivo using a rabbit model of GFS with the insertion of a 24-gauge cannula. A negative control group was included, in the form of the rabbits administered siScramble-hydrogel, which served to isolate the effect on SPARC expression and demonstrate that it is due to siSPARC alone and not other surgical factors. Rabbits administered with MMC were the positive control group, included to compare SPARC to the gold standard treatment used in human surgery. Although instilled as a positive control, a one-time application of MMC at 0.2 mg/ml was unable to prevent bleb loss which had occurred by 2 weeks post-surgery (data not shown), and blebs were no longer visible at 4 weeks (Fig. 5a). Similarly, siScramble-hydrogel treatment failed to maintain bleb survival at 4 weeks (Fig. 5b). In marked contrast, a shallow but still visible bleb can still be observed upon treatment with siSPARC-hydrogel for at least 4 weeks (Fig. 5c).

When the week 4 postoperative tissues were examined by in vivo confocal microscopy, it can be observed that MMC treatment had grossly loosened the subconjunctival matrix with numerous microcysts retained in the failed bleb (Fig. 5d). This morphology contrasted with the densely organized matrix fibers and the lack of microcysts in the subconjunctiva treated with siScramble-hydrogel (Fig. 5e). On the other hand, treatment with siSPARC-hydrogel resulted in an ostensibly perturbed matrix fiber organization and the presence of numerous microcysts reminiscent of MMC treatment, albeit to a lesser extent (Fig. 5f). Moreover, siSPARC-hydrogel treatment was associated with mainly straight vasculature, which included both large and fine vessels in the subconjunctiva (Fig. 5i). This vasculature architecture is a distinguishing feature against the mainly tortuous vessels found in both MMC and siScramble-hydrogel treated tissues (Fig. 5g,h). Finally, while hyper-reflective dots characteristic of MMC treatment were easily visible (Fig. 5j), hydrogel remnants may be visualized within the treated subconjunctivas as a cobbled stone-like network (Fig. 5k,l).

**Figure 6:**
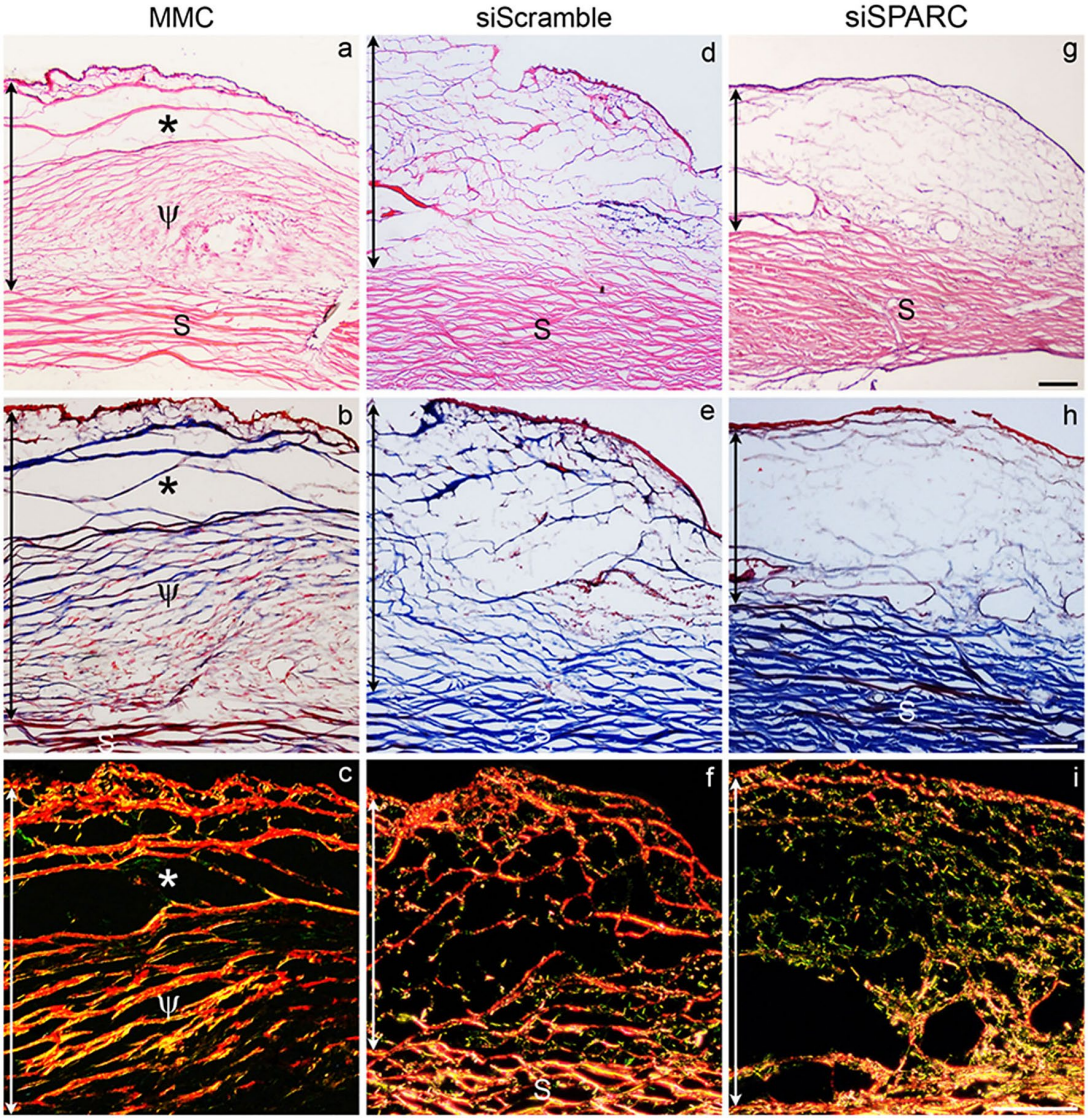
Histological analyses of operated tissues in a rabbit model of glaucoma filtration surgery with insertion of a 24-gauge cannula. (**a**,**d**,**g**) Hematoxylin and eosin (H&E) staining of week 4 postoperative tissues treated with MMC, or hydrogel incorporated with scrambled or SPARC siRNAs. (**b**,**e**,**h**) Masson’s trichrome staining of consecutive sections of the same eyes. (**c**,**f**,**i**) Picrosirius red staining of consecutive sections of the same eyes viewed by polarized microscopy. The vertical double-ended arrow indicates the extent of the subconjunctival matrix, sclera (S). Scale bar represents 100 μm.

Further histological examination of the treated conjunctivas provided corroborating evidence for the effectiveness of the different treatments in facilitating bleb survival. Hematoxylin and eosin (H&E), Masson Trichrome and Picro-Sirius red staining revealed that although a cleared area almost devoid of the matrix was present in the MMC-treated conjunctiva after 4 weeks (*, Fig. 6a–c), a surprisingly relatively larger portion of the tissue was, however, occupied by dense collagen fibers (ψ, Fig. 6a–c). This unusual deposition of collagen scar protein suggests that MMC drug activity was very localized and affected scarring only in areas reached by the administered drug. In contrast, the siScramble-hydrogel treated tissue consisted of less focal deposition of dense collagen fibers, with some areas comprising of disorganized and sparse collagen deposition (Fig. 6d–f). This observation suggests that hydrogel per se may provide a physical barrier to normal scar deposition in the treated area. In both MMC- and siScramble-hydrogel-treated tissues, the collagen fibers present in the operated area were mainly yellowish-orange when visualized using picrosirius red staining (Fig. 6c,f), suggesting that the mature form of collagen had settled in the operated areas by 4 weeks post-surgery. In marked contrast, treatment with siSPARC-hydrogel resulted in a collagen matrix that was vastly distinct from the former two treatments. The collagen matrix in the siSPARC-hydrogel treated area was sparse, diffuse and appeared to consist of seemingly short and disrupted fibers (Fig. 6g,h). Visualization of the sections by picrosirius red staining further revealed that the collagen fibers assembled in response to siSPARC-hydrogel treatment were predominantly greenish-yellow in birefringence (Fig. 6i), suggesting that there may be suppression of maturation of newly-deposited collagen fibers with this treatment.

The data from our rabbit model strongly suggest that our siSPARC-hydrogel treatment can effectively modulate several biological responses associated with bleb survival. Firstly, treatment with siSPARC-hydrogel resulted in straight vasculature and the generation of multiple microcysts, which in humans, have been reported to correspond with a functioning bleb. Secondly, the deposition of diffuse, disrupted and immature collagen fibers resulting from siSPARC-hydrogel treatment is likely to reduce or delay mature scar formation, a major cause of bleb failure. Notably, it appears that the hydrogel component also likely contributed to the mechanical disruption of collagen deposition, further supporting the diffuse morphology that was observed in confocal images and histology. A further advantageous point was the mechanical preservation of a large space within the subconjunctival space by volume of the hydrogel itself, which possibly presented a further obstacle to fibrosis. Overall, the presence of Gtn-Tyr hydrogel allowed the localization and release of siSPARC at the target site as the hydrogel degraded over a specific timeframe, which helps in the bleb survival.

We also compared our siSPARC-hydrogel system with a commonly used anti-scarring agent, MMC. It is well established that the rabbit models demonstrate more intense fibrosis and scar formation and the use of very high dose MMC would be required to maintain bleb survival, which would lead to significant tissue destruction and toxicity^58–62^. Here, the dose which would be used on humans was administered, and scar formation was noted within the first week of surgery. This further cement the importance of targeting SPARC, which in this study was well tolerated. The presence of less subconjunctival scarring led to prolonged bleb survival, and the observed vascularity at week 4 in the siSPARC eyes showed that remodelling was still ongoing. Therefore, future studies will need to be performed to optimise the dose and release of the siRNA in vivo for a more extended period.

## Conclusion

In this work, we demonstrate that a positive-charge tuned gelatin-based hydrogel can safely and effectively deliver siRNA to the cellular environment for efficient gene knockdown. Specifically, we fabricate a Gtn-Tyr hydrogel with both positive-charge tuned and crosslinking properties for siSPARC protection and delivery in vitro and in vivo to demonstrate anti-fibrosis treatment. Our cellular studies involving MTFs show effective and sustained knockdown of SPARC gene and downregulation of Col 1a1. In vivo studies indicate effective inhibition of subconjunctival scarring, after siSPARC delivery using the Gtn-Tyr hydrogel in experimental GFS of ocular scarring using a rabbit model, which is evidenced histologically as well as clinically through bleb morphology. The current charge tunable hydrogel system is a promising delivery platform for safe and effective delivery of siRNAs for efficient gene silencing, and potentially other charged therapeutics.

## Supplementary Information

Supplementary Information.

## Data Availability

The datasets generated during and/or analysed during the current study are available from the corresponding author on reasonable request.
