# Supplementary material for: Positive-charge tuned gelatin hydrogel-siSPARC injectable for siRNA anti-scarring therapy in post glaucoma filtration surgery
Source: Sci Rep. 2021 Jan 14;11:1470. doi: 10.1038/s41598-020-80542-4 (PMC7809290; doi:10.1038/s41598-020-80542-4)
Supplement: Supplementary file 1 — Supplementary Information. [file 41598_2020_80542_MOESM1_ESM.pdf]

Supporting information

Positive-charge tuned gelatin hydrogel-siSPARC injectable for siRNA anti-scarring therapy in post glaucoma filtration surgery

Yong Yao Chun [1,2,+], Zhu Li Yap [3,6,+], Li Fong, Seet [3,4,5], Hiok Hong Chan [6], Li Zhen Toh [3], Stephanie WL Chu [3], Ying Shi Lee [3,6], Tina T. Wong [3,4,5,6,7,*], Timothy T. Y. Tan [2,*]

[1] Ocular Imaging, Singapore Eye Research Institute, 20 College Road Discovery Tower Level 6, The Academia, Singapore 169856.
[2] School of Chemical and Biomedical Engineering, Nanyang Technological University, 62 Nanyang Dr, Singapore 637459.
[3] Ocular Therapeutics and Drug Delivery, Singapore Eye Research Institute, 20 College Road Discovery Tower Level 6, The Academia, Singapore 169856.
[4] Department of Ophthalmology, Yong Loo Lin School of Medicine, National University of Singapore, 10 Medical Dr, Singapore 117597.
[5] Duke-NUS Medical School, 8 College Rd, Singapore 169857.
[6] Glaucoma Service, Singapore National Eye Centre, 11 Third Hospital Ave, Singapore 168751.
[7] School of Materials Science and Engineering, Nanyang Technological University, 11 Faculty Ave, Singapore 639977.

*Corresponding authors
Timothy Tan (tytan@ntu.edu.sg)
Tina T Wong (tina.wong.t.l@singhealth.com.sg)

[+]Yong Yao Chun and Zhu Li Yap contributed equally to this work.

Stability assay of siSPARC treated with H_2O_2

siSPARC (100 pmol/ul) was treated with 3mM H_2O_2 and its stability was compared with naked siSPARC as control. After the treatment, the siSPARC was analyzed using 5 % native agarose gel electrophoresis. The 5 % agarose gel was prepared by dissolving 5 g of agarose powder (Vivantis Technologies Sdn Bhd, Malaysia) in 100 ml of 1 × Tris-Acetate-EDTA (TAE) buffer (1st Base, Sinagpore). The mixture was microwave for 1-3 min until the agarose is completely dissolved. 10 ul SYBR Safe (Invitrogen, US) was then added to the agarose solution for staining of nuclei acid. The mixture was then poured into a gel tray with the well comb and allowed to set. siSPARC sample was mixed DNA Gel Loading Dye, Blue (6 ×; Thermo Fisher Scientific, US) and loaded onto the agarose gel with 1 nmol per lane. The gel electrophoresis was performed at 80V for 50 minutes (Bio-Rad, US) and visualized using blue LED transilluminator (Beijing Biopeony Co. Ltd, China).

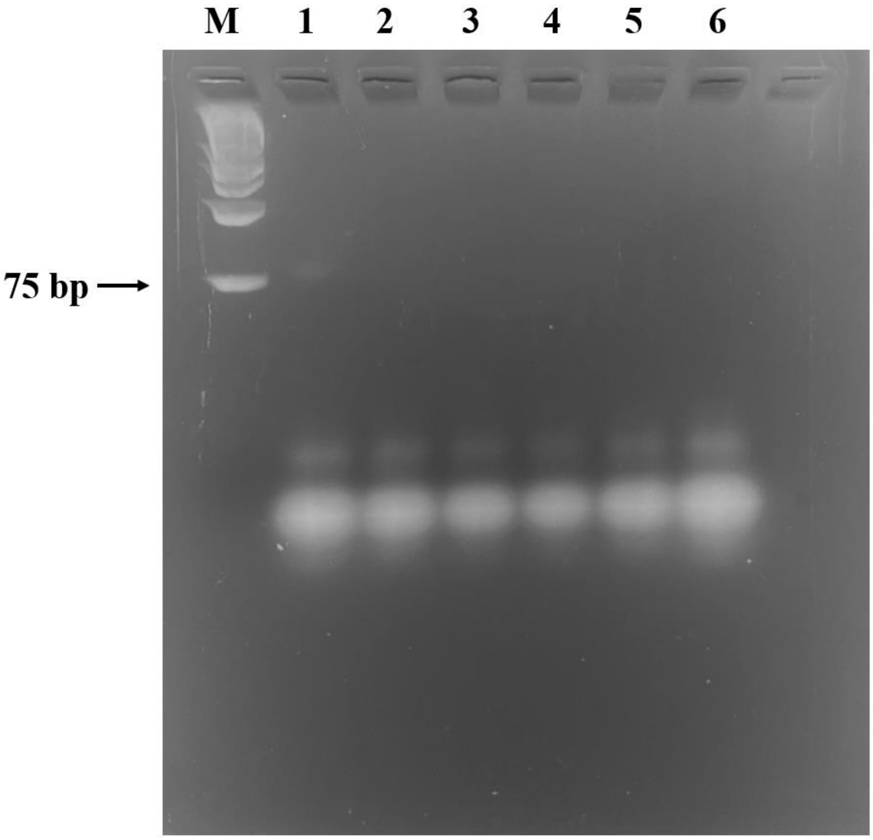

Figure S1. Nucleic acid electrophoresis on 5 % agarose gel at 80 V for 50 min. GeneRuler TM 1 kb Plus DNA markers (M, in bp; Thermo Fisher Scientific, US), siSPARC treated with 3 mM H_2O_2 (1-3) and naked siSPARC (4-6) as control.

Rheological analysis

Gtn-Tyr hydrogel was fabricated using 0.12 units/ml HRP and 3 mM H_2O_2 and cast into a mould. Cylinder samples with 8 mm diameter and 2 mm thickness were then cut out for rheological analysis. Storage modulus (G') of the sample was determined using rheometer MCR 501 (Anton Paar, Austria) with probe PP08/150. The measurement was performed using oscillation mode with a constant frequency of 1 Hz at 37 ^{o}C.

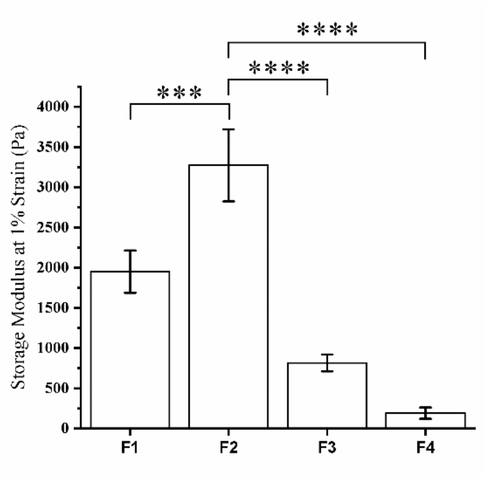

Figure S2. Storage modulus at 1 % strain of Gtn-Tyr hydrogels with increasing zeta potential (F1 to F4). *** denote $P < 0.001$ and **** denote $P < 0.0001$.

***In vitro* degradation studies**

5 w/v% Gtn-Tyr solution was prepared by dissolving freeze-dried precursor into 1× PBS. HRP and H_2O_2 with a final concentration of 0.12 unit/ml and 3 mM respectively were added to 0.5 ml precursor solution in a 2 ml microtube to initiate the crosslinking process. The sample was allowed to set for 0.5 h before topping up with 0.5 ml collagenase type I (Gibco, Life Technologies, US) with a concentration of 0.5 units/ml. At a designated time, the collagenase solution was removed, and the sample weighed before replenished with fresh collagenase solution. The percentage weight loss at each time point was calculated using the formula below:

$$\text{Percentage weight loss } (\%) = \frac{W_i - W_t}{W_i} X \ 100\%$$

Where W_i and W_t were the sample's initial weight and weight at the designated time, respectively.

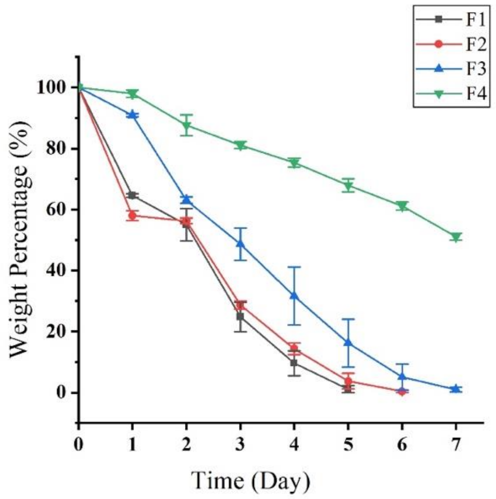

Figure S3. Hydrogel degradation with different surface charge.

Investigation of electrostatic interaction between Gtn-Tyr hydrogel and siSPARC using zeta potential measurement

Gtn-Tyr precursor was fabricated following the "**Materials and methods: Synthesis of Gtn-Tyr precursor"** and using 57.6 mM tyramine chloride (Tyr.Cl; Sigma-Aldrich, US), 10.6 mM N-(3-dimethylaminopropyl)-N′-ethylcarbodiimide hydrochloride (EDC.HCl; Sigma-Aldrich, US) and 5.3 mM N-hydroxysuccinimide (NHS; Sigma-Aldrich, US). The freeze-dried Gtn-Tyr precursor was dissolved in DI water with a concentration of 5 w/v%. siSPARC was then added into the Gtn-Tyr solution to have a final concentration of 4 nmol/ml and allowed to interact for 15 min. The zeta potential of Gtn-Tyr without and with siSPARC was measured in the DTS1070 disposable folded capillary cells at the temperature of 25 ^{o}C. The instrument was calibrated using latex with known zeta potential. Each sample was tested three times, and the average of 3 different samples prepared using the same condition was calculated.

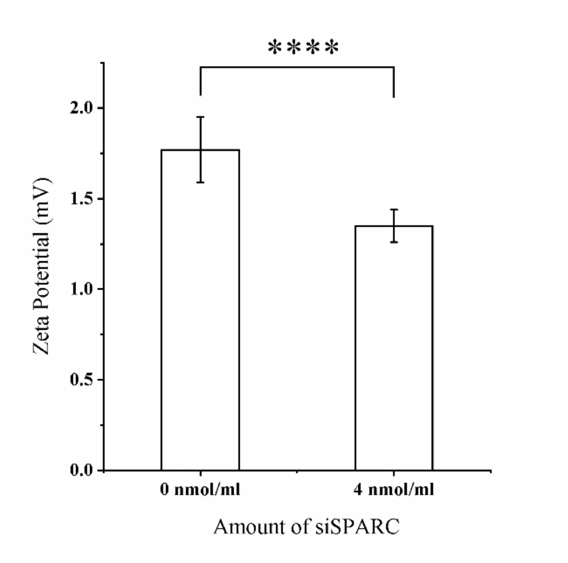

Figure S4. Graph showing zeta potential of Gtn-Tyr hydrogel with + 1.77 ± 0.18 mV was significantly reduced ($P < 0.0001$) to + 1.35 ± 0.09 mV after the interaction with 4 nmol/ml siSPARC. **** denote $P < 0.0001$.

Size measurement of Gtn-Tyr hydrogel degradation products

5 w/v% Gtn-Tyr solution was prepared by dissolving freeze-dried precursor into 1× PBS. HRP and H_2O_2 with a final concentration of 0.15 unit/ml and 3 mM respectively were added to 0.5 ml precursor solution to initiate the crosslinking process. The mixture was poured into 24 well plate and allow to set. Next, 0.5 ml collagenase type I (1.0 units/ml) was added to the Gtn-Tyr hydrogel to digest the sample. After 1-day treatment, the solution was characterized using the Malvern Zetasizer (Malvern Panalytical Ltd, UK) to measure the size of degraded products. Next, the size of the optimum formulation (F2) interacted with 4 nmol/ml siSPARC and degraded products of siSPARC-hydrogel was also measured.

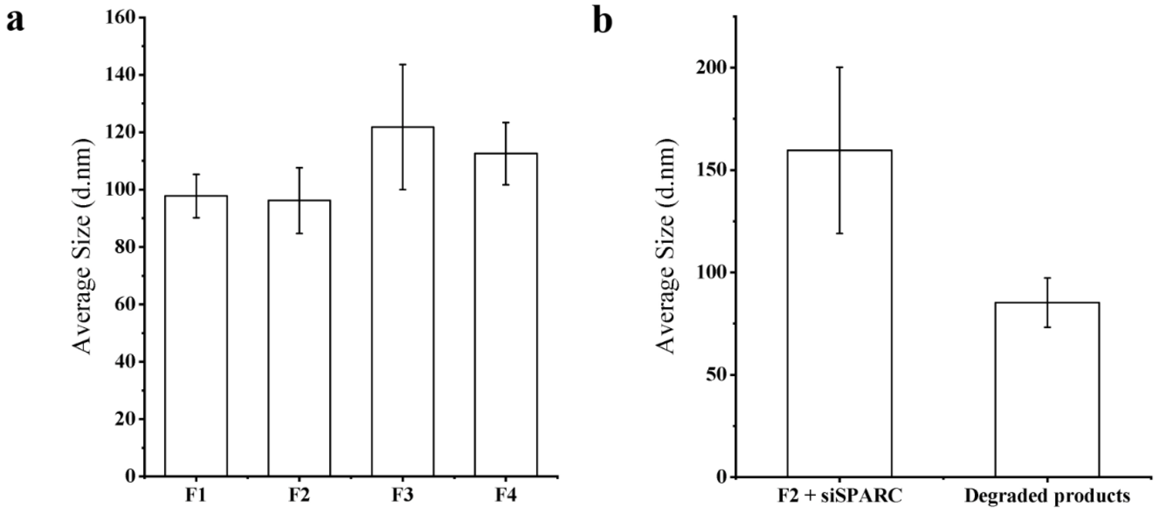

Figure S5. Average size (d.nm) of (a) the degraded products of Gtn-Tyr hydrogel synthesized with different formulations after 1-day treatment using 1.0 units/ml collagenase type I, (b) optimum formulation (F2) with 4 nmol/ml siSPARC and degraded products of siSPARC-hydrogel.

***In vitro* SPARC silencing of C57Bl6/J mouse tenon fibroblasts (MTFs) using the optimum formulation (F2) with different amount of siSPARC**

Freeze-dried F2 was dissolved with DMEM high glucose without FBS and loaded with 1, 2 or 4 nmol/ml siSPARC. Next, 0.5 ml F2 hydrogel containing siSPARC was fabricated by adding HRP and H_2O_2 with a final concentration of 0.15 units/ml and 3 mM respectively. The mixture was then cast onto a 12 well plate. C57Bl6/J MTFs were seeded on top of the hydrogel with a density of 3×10^4 cells per well. The sample was topped up with 0.5 ml culture medium and incubated at 37 °C and 5 % CO_2. At day 2 and 7, total RNA was recovered with Trizol Reagent (Invitrogen Corp., US) according to the manufacturer's recommendations. First-strand cDNA was synthesized with 500 ng total RNA extract and 1 µl of 50 ng/µl random hexamer primer (Invitrogen Corp., US) with Superscript III reverse transcriptase (Invitrogen Corp., US) according to the manufacturer's instructions. Quantitative real-time PCR (qPCR) was performed in a total volume of 10 µl in 384-well microtiter plates. Each reaction consisted of 1 µl of the first-strand reaction product, 0.5 µl each of upstream and downstream primers (10 µM each), 4 µl of Power SYBR Green PCR Master Mix (Applied BioSystems, US), and 4 ul of DNase-RNase-free distilled water (Sigma-Aldrich, US). Amplification and analysis of cDNA fragments were carried out by use of the Roche LightCycler 480 System (Roche Diagnostics Corp, US). All PCR reactions were

performed in triplicate. All mRNA levels were measured as CT threshold levels and were normalized with the corresponding 18S CT values (housekeeping gene). Values are expressed as fold increase over the corresponding values for untreated WT control by the 2−ΔΔCT method.

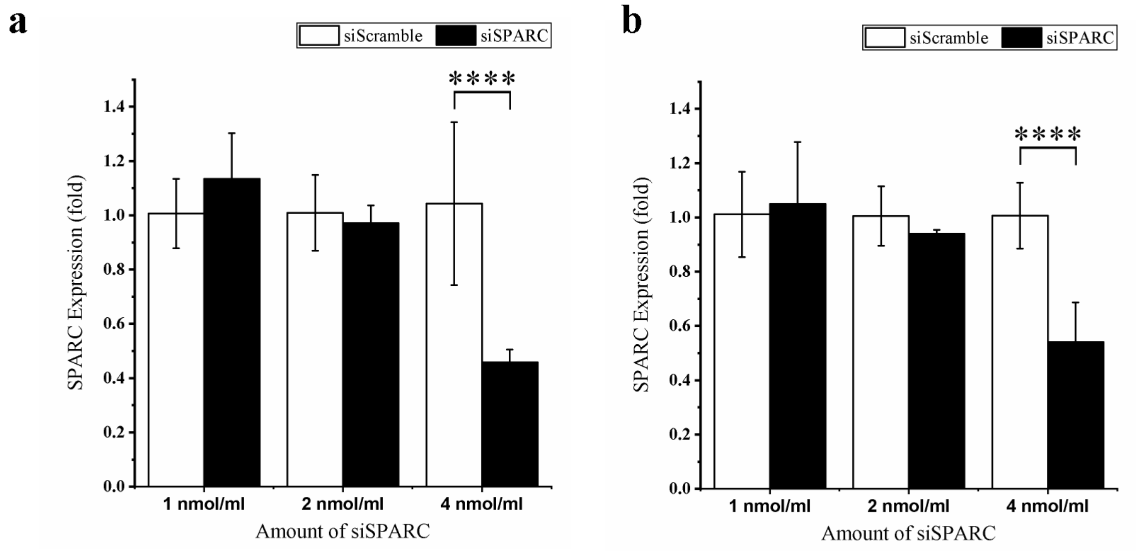

Figure S6. SPARC gene expression of MTFs after treated with F2 hydrogel with 1, 2 or 4 nmol/ml siScramble or siSPARC after (a) day 2 and (b) day 7. **** denote $P < 0.0001$.

Western blot

After treated with Gtn-Tyr hydrogel containing 1 or 4 nmol/ml siSPARC or siScramble for 7 days, C57Bl6/J MTFs was extracted and lysed using a solution containing 20 mM Tris buffer (pH 7.4), 150 mM NaCl, 1 mM EDTA, 0.5% Triton X-100, 2 mM $MgCl_2$, 1 mM dithiothreitol, and 1× Complete Protease Inhibitors (Roche Diagnostics GmbH, Germany). SDS-polyacrylamide gel electrophoresis and immunoblotting were performed using the antibodies against SPARC, β-tubulin, GAPDH (Santa Cruz Biotechnology, Inc., US), collagen I (Novus Biologicals, US) and horseradish peroxidase (HRP)–conjugated secondary antibodies (Jackson Immunoresearch Laboratories, Inc., US). Densitometric quantitation was performed according to a previously established protocol[1], and potential errors in loading were corrected to levels of GAPDH (housekeeping protein).

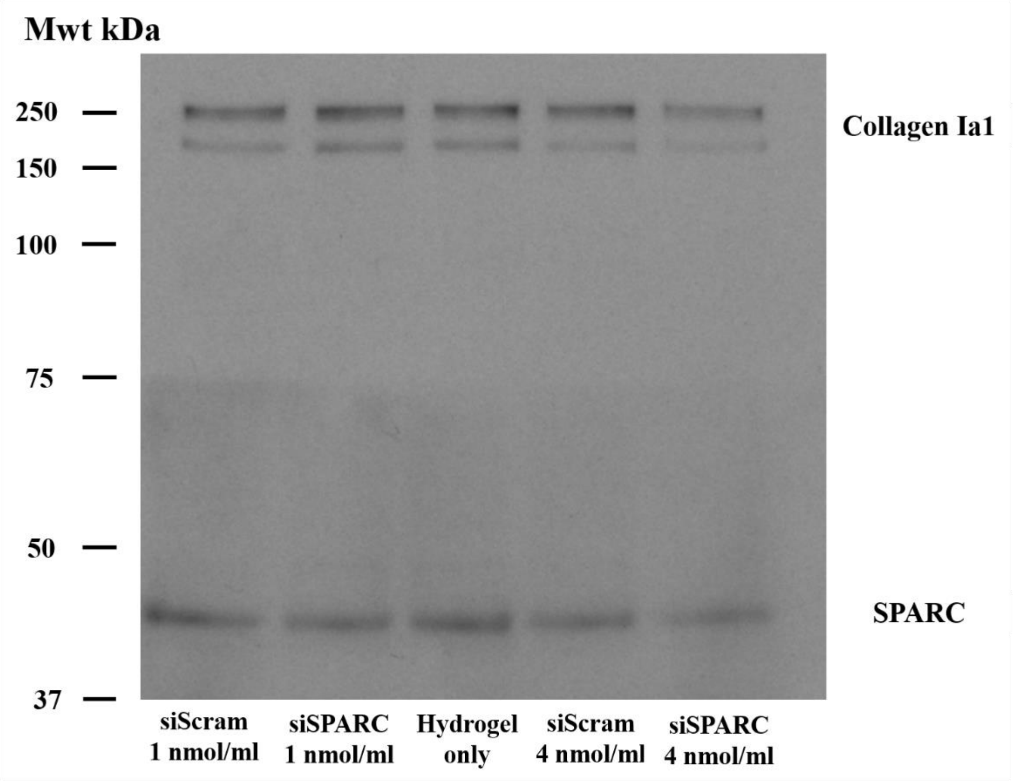

Figure S7. Representative western blot image showing the proteins SPARC and Collagen Ia1 for MTFs after treated with Gtn-Tyr hydrogel containing 0, 1, and 4 nmol siScramble or siSPARC for 7 days.

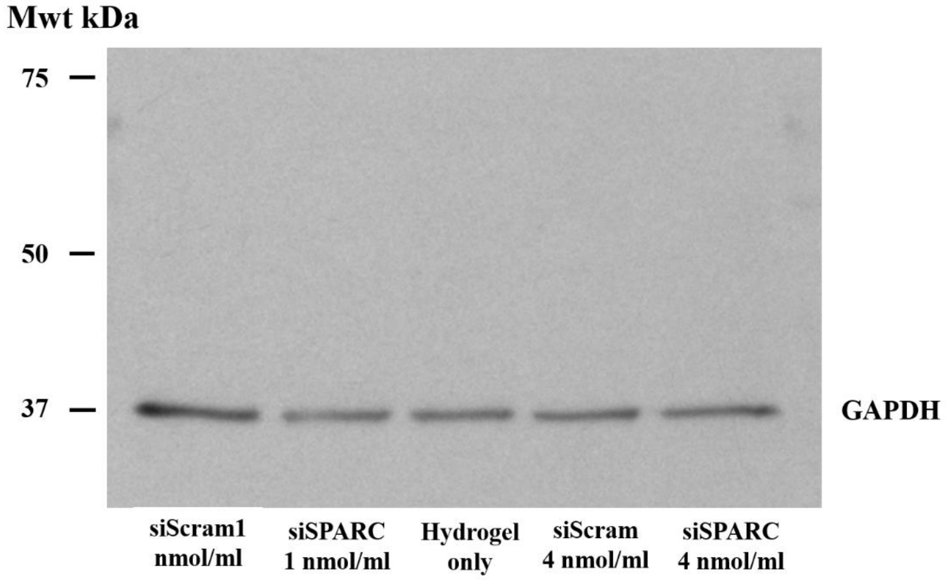

Figure S8. Representative western blot image showing the housekeeping protein, GADPH, for MTFs after treated with Gtn-Tyr hydrogel containing 0, 1, and 4 nmol/ml siScramble or siSPARC for 7 days.

MTF's proliferation and viability studies

Freeze-dried Gtn-Tyr (F2) was dissolved with DMEM high glucose without FBS (5 w/v%). The hydrogel precursor was sterilized using a 0.22μm syringe filter. Next, 0.5 ml Gtn-Tyr hydrogel was fabricated by adding HRP and H_2O_2 with a final concentration of 0.15 units/ml and 3 mM, respectively. The mixture was then cast onto a 24 well plate. C57Bl6/J MTFs were seeded on top of the hydrogel with a density of 2×10^4 cells per well. The sample was topped up with 0.5 ml culture medium and incubated at 37 ^oC and 5 % CO_2. At day 1, 3 and 7, the cell proliferation rate was measured using PrestoBlue cell viability reagent (Life Technologies, US). LIVE/DEAD cell viability assay (LifeTechnologies, US) was used to observe live and dead cells cultured on the hydrogel at day 1, 3 and 7 using the Zeiss Axio Observer Z1 inverted microscope (Carl Zeiss, Germany).

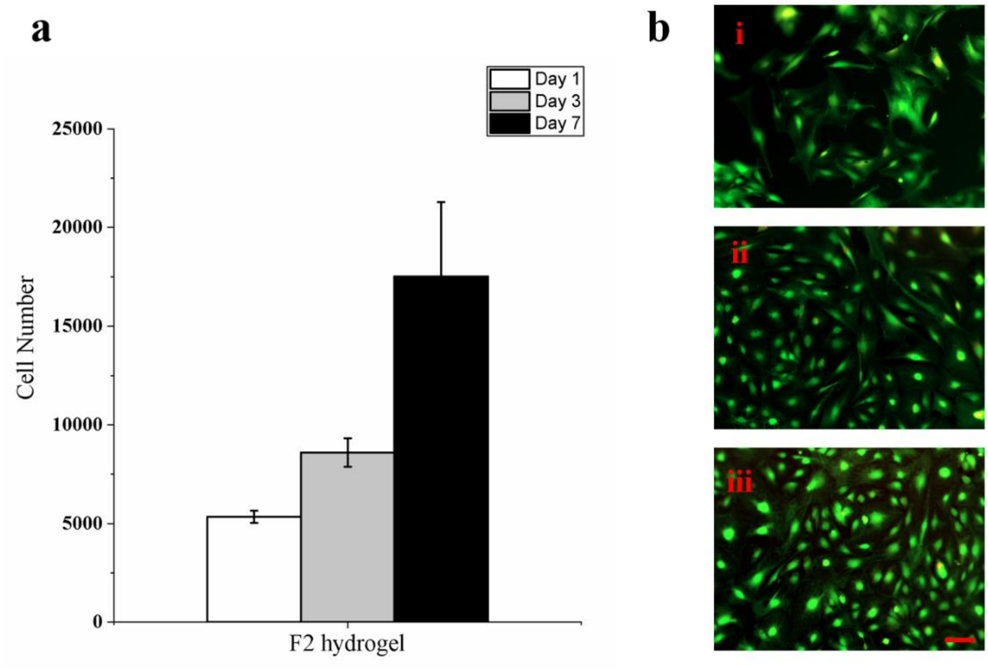

Figure S9. Graph on (a) cell proliferation of MTFs cultured on F2 hydrogel using the PrestoBlue assay and visual observation of (b) cell viability at day (i) 1, (ii) 3 and (iii) 7 through LIVE/DEAD cell viability assay (green fluorescence represents live cells; red fluorescence represents dead cells; scale bar represents 100 μm).

Reference

1. Seet, L.-F. *et al.* SPARC deficiency results in improved surgical survival in a novel mouse model of glaucoma filtration surgery. *PloS one* **5**, e9415 (2010).
